# Assessment of blood clot formation in patients with Primary Sjögren’s syndrome (pSS)

**DOI:** 10.1186/1471-2474-14-S1-A15

**Published:** 2013-02-14

**Authors:** K Collins, K Balasubramaniam, B Griffiths, G Viswanathan, A Natasari, J Tarn, A Zaman, WF Ng

**Affiliations:** 1 Musculoskeletal Research Group, Institute of Cellular Medicine, Newcastle University, UK; 2 Freeman Hospital, Newcastle upon Tyne, UK

## Background

Primary Sjögren’s syndrome (pSS) is an autoimmune rheumatic disease affecting the exocrine glands. Several pSS-associated clinical and laboratory parameters may predispose to thromboembolic risk, however clinical data is conflicting.

My primary objective was to test whether whole blood clot formation is abnormal in pSS using several complementary approaches: Thromboelestrography (TEG) and Multiplate platelet mapping (MPP) for pSS patients compared to healthy and disease (lupus-SLE) controls and compare the size/composition of blood clots formed ex-vivo between pSS patients and healthy controls using a clotting chamber, which simulates the clotting process in-vivo.

My secondary objectives were to determine if any clinical/laboratory/cytokine parameters were associated with abnormal clotting.

## Methods

TEG and MPP were performed on blood samples from 12 healthy controls, 24 pSS and 11 SLE patients.

For TEG: clot formation speed, strength and lysis rate were analysed. For MPP, platelet receptor responses to common agonists were investigated.

12 pSS patients completed a clotting chamber procedure.

Univariate correlation analysis was used to determine the relationship between clotting and clinical/laboratory/cytokine parameters.

## Results

All TEG and MPP parameters were similar between groups. Statistically significant correlations were found for (a) ESR and clot lysis parameters (p<0.007); (b) CD40L and clot formation/lysis rate (P<0.01).

Analysis of clot specimens from the clotting chamber is on-going. Preliminary data indicate that several cytokines are “consumed” during the clot formation.

## Discussion

There was no difference in clotting and platelet receptors function between pSS patients and lupus patients/healthy controls. Interestingly, several laboratory/clinical parameters significantly correlate with clotting/platelet receptor function.

